# Distinct genetic changes reveal evolutionary history and heterogeneous molecular grade of DLBCL with *MYC*/*BCL2* double-hit

**DOI:** 10.1038/s41375-019-0691-6

**Published:** 2019-12-16

**Authors:** Francesco Cucco, Sharon Barrans, Chulin Sha, Alexandra Clipson, Simon Crouch, Rachel Dobson, Zi Chen, Joe Sneath Thompson, Matthew A. Care, Thomas Cummin, Josh Caddy, Hongxiang Liu, Anne Robinson, Anna Schuh, Jude Fitzgibbon, Daniel Painter, Alexandra Smith, Eve Roman, Reuben Tooze, Catherine Burton, Andrew J. Davies, David R. Westhead, Peter W. M. Johnson, Ming-Qing Du

**Affiliations:** 1 Department of Pathology, University of Cambridge, Cambridge, UK; 2 Haematological Malignancy Diagnostic Service, St James’ University Hospital, Leeds, UK; 3 Faculty of Biological Sciences, University of Leeds, Leeds, UK; 4 Department of Health Sciences, University of York, York, UK; 5 Cancer Research UK Centre and Southampton Clinical Trials Unit, University of Southampton, Southampton, UK; 6 Haematopathology and Oncology Diagnostics Service, Cambridge University NHS Foundation Trust, Cambridge, UK; 7 Department of Oncology, University of Oxford, Oxford, UK; 8 Centre for Haemato-Oncology, Barts Cancer Institute, London, UK

**Keywords:** Cancer genetics, Clinical genetics

## Abstract

Using a Burkitt lymphoma-like gene expression signature, we recently defined a high-risk molecular high-grade (MHG) group mainly within germinal centre B-cell like diffuse large B-cell lymphomas (GCB-DLBCL), which was enriched for *MYC*/*BCL2* double-hit (*MYC*/*BCL2*-DH). The genetic basis underlying MHG-DLBCL and their aggressive clinical behaviour remain unknown. We investigated 697 cases of DLBCL, particularly those with *MYC*/*BCL2*-DH (*n* = 62) by targeted sequencing and gene expression profiling. We showed that DLBCL with *MYC*/*BCL2*-DH, and those with *BCL2* translocation, harbour the characteristic mutation signatures that are associated with follicular lymphoma and its high-grade transformation. We identified frequent *MYC* hotspot mutations that affect the phosphorylation site (T58) and its adjacent amino acids, which are important for MYC protein degradation. These *MYC* mutations were seen in a subset of cases with *MYC* translocation, but predominantly in those of MHG. The mutations were more frequent in double-hit lymphomas with IG as the *MYC* translocation partner, and were associated with higher MYC protein expression and poor patient survival. DLBCL with *MYC*/*BCL2*-DH and those with *BCL2* translocation alone are most likely derived from follicular lymphoma or its precursor lesion, and acquisition of *MYC* pathogenic mutations may augment MYC function, resulting in aggressive clinical behaviour.

## Introduction

Diffuse large B-cell lymphoma (DLBCL) is the most common lymphoma in adults, accounting for around 75% of aggressive lymphomas. The current standard treatment for DLBCL is immunochemotherapy, typically R-CHOP (rituximab plus cyclophosphamide, doxorubicin, vincristine and prednisone), with 60% of patients living 10 years or more [1]. Patients who fail R-CHOP treatment respond poorly to currently available alternative therapies, and mortality is highest within the first two years after diagnosis [1]. The ability to risk-stratify patients with a low probability of cure following R-CHOP at diagnosis, and to enter them into clinical trials investigating alternative therapies is a significant unmet clinical need. A number of biomarkers have been investigated, but only *MYC* translocation and to a lesser extent, cell of origin (COO), are used routinely or in a clinical trial [1].

*MYC* translocation occurs in ~10% of DLBCL, and is frequently (21–83%) accompanied by an additional *BCL2* and/or *BCL6* translocation, known as double-hit (DH) or triple-hit (TH) [2–15]. *MYC/BCL2*-DH DLBCL are generally aggressive and respond poorly to currently available therapies, with the majority of patients dying within two years of diagnosis, although a minority of cases experience a long term survival [15, 16]. The clinical outcomes for *MYC*/*BCL6*-DH DLBCL are less clear owing to the small number of cases investigated and their heterogeneous COO [14, 17, 18]. Patients with a single *MYC* translocation (*MYC*-SH) show variable clinical courses. A subset of these cases has *TP53* mutations and displays a worse survival, similar to that of *MYC/BCL2*-DH [18, 19]. There remains a need to clarify the prognostic stratification of *MYC* translocation-bearing DLBCL.

Based on COO classification, DLBCL has been divided into two broad subgroups: activated B-cell like (ABC) and germinal centre B-cell like (GCB) subtypes, with the ABC-DLBCL showing enhanced NF-κB activation and generally a worse prognosis [20, 21]. Although the COO classification provides broad biologically distinct categories, there is an apparent heterogeneity in clinical outcome within each subtype. Further subclassification of these broad molecular subtypes has been investigated by several recent studies based on clustered genetic changes and/or gene expression signatures [22–26]. Among these recent advances, Sha et al. and Ennishi et al. have defined a clinically and biologically distinct high-risk subgroup within GCB-DLBCL using, respectively, a Burkitt lymphoma-like or *MYC*/*BCL2*-DH-founded gene expression signature [25–27]. This subgroup, termed molecular high-grade (MHG) in the study by Sha et al., is enriched in cases with *MYC*/*BCL2*-DH, and more importantly includes other poor prognosis cases without the double-hit, which are not readily identified by other methods [25, 28].

Intriguingly, although MHG-DLBCL is enriched in *MYC*/*BCL2*-DH, a proportion of cases with *MYC*/*BCL2*-DH do not have the MHG gene expression signature and these cases show no worse survival than that of conventional GCB-DLBCL (non-MHG) [25]. To understand the genetic basis underlying MHG-DLBCL and its aggressive clinical behaviour, we used targeted sequencing to investigate 697 cases of DLBCL, particularly enriched for those with *MYC*/*BCL2*-DH(TH) (*n* = 62).

## Methods

### Case selection and materials

Of the 697 DLBCL cases included, 400 were from the REMoDL-B trial (28 *MYC* translocation positive) and 297 (97 *MYC* translocation positive) from a UK population-based cohort [25, 28]. The vast majority of cases in the population-based cohort were from the Haematological Malignancy Research Network (HMRN) (www.hmrn.org), which tracks all haematological malignancies across 14 hospitals diagnosed by a centralised fully-integrated haematopathology laboratory [29]. Case selection in the present study was biased towards those with a *MYC* translocation, which, together with *BCL2* and *BCL6* translocations, was determined at the time of diagnosis (HMRN) or retrospectively collected from pathology records or by performing interphase fluorescent in situ hybridisation (FISH) on TMA (REMoDL-B) [18, 25]. Among the population-based cohort, five cases had a previous history of follicular lymphoma (FL), and a further 28 cases had a histological evidence of concurrent FL. For the REMoDL-B trial, patients with a previous history of low-grade lymphoma were excluded.

Formalin-fixed paraffin-embedded (FFPE) diagnostic tissue biopsy was available in each case and local ethical guidelines were followed for the use of these tissue materials for research with the approval of the ethics committees of the involved institutions (05-Q1604–10, 04-Q1205–125, 10-H0504–79).

### DNA extraction and quality assessment

Haematoxylin and eosin stained FFPE tissue slides were reviewed and tumour rich areas (>40%) in consecutive sections were isolated by crude macrodissection in each specimen. DNA was extracted using the QIAamp DNA Micro Kit (QIAGEN, Crawley, UK) and quantified using a Qubit® Fluorometer (Life Technologies, UK). The quality of DNA samples was assessed by PCR of variably sized genomic fragments using a standardised protocol [30].

### Targeted sequencing by HaloPlexHS enrichment and Illumina HiSeq sequencing

This was essentially carried out as described previously [31]. Briefly, 100 ng genomic DNA was subjected to targeted enrichment of 70 genes (Table S1), which are recurrently mutated in aggressive B-cell lymphomas using a customised HaloPlexHS probe library (Agilent Technologies). The HaloPlexHS probes incorporate molecular barcodes, hence allowing removal of PCR errors during data analysis. Library preparation was performed according to the manufacturer’s instructions for FFPE tissue samples. The pooled libraries were sequenced on an Illumina HiSeq4000 (2 × 150 bp end sequencing protocol) or HiSeq2500 (Rapid Run Mode 2 × 150 bp end sequencing protocol). As stipulated by our previous study, DNA samples amenable for PCR of ≥400 bp genomic fragments were investigated in a single replicate, while those amenable for PCR of 300 bp were analysed in duplicates, with reproducible variants being considered as a true change [31].

### Variant calling and annotation

Sequence data analysis and variants calling were performed using a previously validated in-house protocol [31]. Briefly, SNV were called using UnifiedGenotyper with no downsampling [32]. As this was unable to call SNVs at <8% AAF reliably, MuTect2 was additionally employed for detection of hotspot mutations at low AAF values. Indel detection was separately carried out on the recalibrated bam files using Pindel v0.2.5 [33], which allowed detection of indels as low as 2% AAF. Variant calling files were concatenated to produce one library vcf each for the SNV and Indel pipelines, and then filtered using vcftools v0.1.15 and bedtools v2.25 for read depth, quality score and known PCR/sequence artefacts. Further filtering was carried out to remove variants in intronic regions outside essential splicing sites, SNPs with a minor allele frequency ≥0.1% (dbSNP database, 1000 Genomes Project, the ExAC exome sequencing database) and synonymous changes. In addition, missense variants predicted to be benign by at least seven out of nine functional prediction tools (SIFT, Polyphen2 HDIV, Polyphen2 HVAR, LRT, MutationTaster, MutationAssessor, FATHMM, SVM score and LR score) were excluded. The resulting variants were further scrutinised by reviewing the bam file to eliminate potential PCR and sequence artefacts. Only variants above the cut-off value (20 alternative allele depth for DNA samples amenable for PCR of ≥400 bp, 15 alternative allele depth in both replicates for DNA samples amenable for PCR of 300 bp) were considered to be a true change [31]. Finally, extensive search of COSMIC database and published literature was carried out to retain those known and confirmed to be somatic variants. The final mutation list can be found in Table S2.

### Molecular subtyping by gene expression profiling

Whole-genome gene expression profiling was performed on mRNA extracted from FFPE diagnostic tissue specimens using the Illumina whole-genome DASL array [34]. Data analyses and COO classification were carried out using the “DLBCL automatic classifier” (DAC) [35]. The MHG group was identified based on a Burkitt lymphoma-like signature as defined in previous studies [25, 36].

### Interphase fluorescence in situ hybridisation (FISH)

Chromosome translocation status was available from routine haematopathological diagnosis or previous studies for *MYC*, *BCL2* and *BCL6* in 550, 233 and 218 cases, respectively [18, 25]. In the REMoDL-B and HMRN cohort, *MYC* translocation was screened with Dako *MYC* break-apart probe, and those showing no evidence of *MYC* translocation but with MHG phenotype were further investigated with Abbott *MYC* break-apart and *MYC*/*IGH* dual fusion probe. In the remaining cases from other UK hospitals, *MYC* translocation was investigated with Abbott *MYC* break-apart probe.

Interphase FISH was performed for *BCL2* and *BCL6* translocation in 433 and 366 cases in the present study. In cases with *MYC* translocation, additional FISH was performed with *MYC*/*IGH* (Abbott) (if not yet done), then *MYC*/*IGK* and *MYC*/*IGL* (Cytocell) dual fusion probes in those without any evidence of *MYC* and *IGH* fusion. All FISH was carried out on tissue microarray or whole tissue section as described previously [18, 25].

### MYC immunohistochemistry

Data on MYC protein expression by immunohistochemistry on tissue microarrays (TMA) slides were available from our previous study [25]. The immunostained slides were scanned and MYC protein expression was quantified and presented as percentage of positive nuclear staining in lymphoma cells using IHC Profiler Image-J software according to the instructions for nuclear protein targets (https://imagej.net/) [37].

### Statistical analysis

The probability of *MYC* hotspot mutations occurring by chance was assessed by Poisson regression. Associations among chromosomal rearrangements, mutations and clinical variables were analysed using Fisher’s exact test. Survival analysis was carried out using Cox proportional hazards and likelihood ratio tests in R (https://cran.r-project.org). All quoted *P* values are two-sided.

## Results

**Fig. 1:**
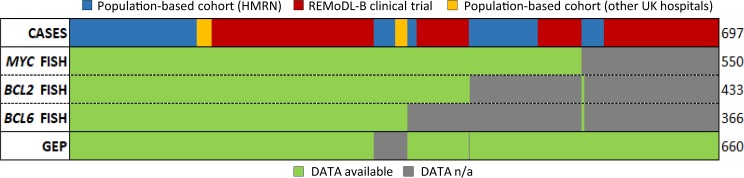
Summary of cases of DLBCL included in the study. A total of 697 cases were studied, including 400 from the REMoDL-B trial and 297 cases from population-based cohort, mainly from the Haematological Malignancy Research Network (HMRN). Laboratory data on chromosome translocations and molecular subtypes by gene expression profiling are indicated.

**Fig. 2:**
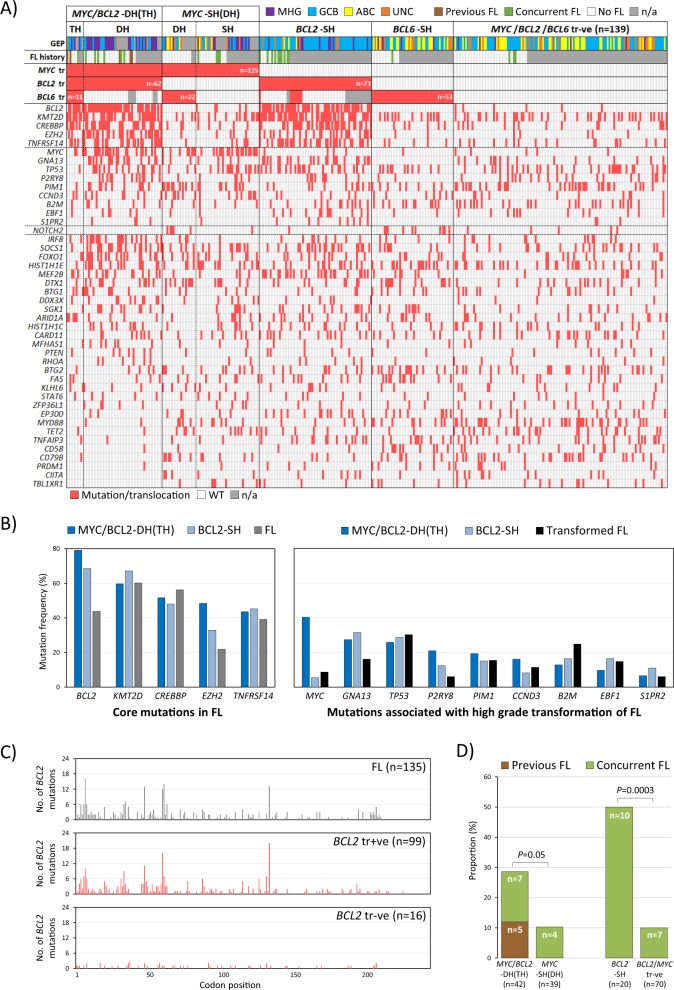
DLBCL with *BCL2* translocation harbour the cardinal mutation signature of follicular lymphoma. **a** Heatmap illustration of mutation distribution according to chromosome translocation status; Where data available, evidence of previous or concurrent follicular lymphoma is indicated. **b** DLBCL with *BCL2* translocation, particularly those with *MYC*/*BCL2*-DH, harbour the cardinal mutation signature of FL, and also the mutation profile associated with its high-grade transformation [38–41]. Representative mutation data in FL and transformed FL are from the study by Kridel et al. [41], with *EZH2* mutation considered as the core changes associated with FL [38–41]. **c** Comparison of *BCL2* mutation profile between *BCL2* translocation positive DLBCL in the present study and FL in the study by Huet et al. [42]. **d** DLBCL with *BCL2* translocation often have a previous or concurrent follicular lymphoma. *MYC*/*BCL2*-DH *MYC*/*BCL2* double-hit, TH *MYC*/*BCL2*/*BCL6* triple-hit, SH single-hit, tr +ve/−ve translocation positive/negative, FL follicular lymphoma.

Among the 697 cases investigated by targeted sequencing, 553 were investigated for chromosomal translocations by interphase FISH; and *MYC, BCL2* and *BCL6* translocations were found in 125, 136 and 97 cases, respectively, with *MYC*/*BCL2*/*BCL6*-TH in 11, *MYC*/*BCL2*-DH in 51 (*BCL6* translocation data unknown in 8) and *MYC*/*BCL6*-DH in 22 (Figs. 1 and 2).

### The mutation profile of DLBCL with *MYC/BCL2*-DH *or BCL2-*SH suggests their derivation from follicular lymphoma

In general, DLBCL with *MYC*/*BCL2/BCL6*-TH and *MYC*/*BCL2*-DH had a similar mutation profile, and were characterised by a higher mutation load and more frequent mutations in *BCL2*, *KMT2D*, *CREBBP*, *EZH2* and *TNFRSF14* than those with isolated *MYC* translocation (Figs. 2a,  S1 and S2). Interestingly, these mutations are the cardinal features of FL [38–42], and were similarly seen in DLBCL with *BCL2*-SH (Fig. 2b). The *BCL2* mutation profile was almost identical between *BCL2* translocation positive DLBCL and FL (Fig. 2c) [42]. These findings suggest that DLBCL with a *BCL2* translocation may be derived from a FL or its precursor lesion. In support of this suggestion, DLBCL with *MYC*/*BCL2*-DH(TH) and those with *BCL2*-SH also harboured an additional mutation profile (*MYC*, *GNA13*, *TP53*, *P2RY8, PIM1*, *CCND3*, *B2M, EBF1* and *S1PR2*), which was associated with high-grade transformation of FL as shown by several previous studies (Fig. 2b) [38–41]. Furthermore, both groups (28 and 50%, respectively) frequently presented with either a previous or concurrent FL (Fig. 2d). Intriguingly, DLBCL with *MYC*-*BCL2*-DH(TH) were more often associated with a previous, but not concurrent FL than those with *BCL2*-SH. Nonetheless, 65% of BCL2 translocation positive cases lacked documented evidence of FL at diagnosis (Fig. 2d). With the exception of *MYC* mutations, there was no significant difference in the mutation profile between *BCL2* translocation positive cases with and without FL (Fig. S3). *MYC* mutation was more frequent in BCL2 translocation positive cases without FL, and this was primarily due to a high frequency of *MYC* translocation in this group.

**Fig. 3:**
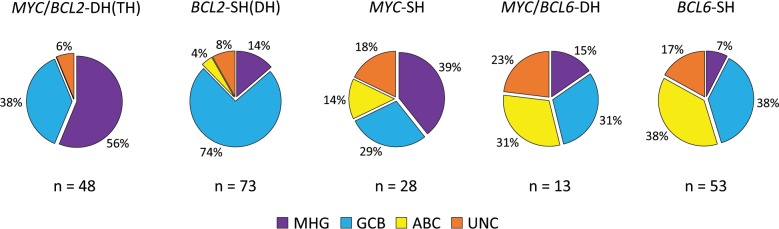
Molecular subtype of DLBCL according to translocation status. MHG molecular high-grade, GCB germinal centre B-cell like, ABC activated B-cell like, UNC unclassified.

As expected, DLBCLs with *MYC/BCL2*-DH(TH) were either MHG (56%) or GCB (38%), the remaining cases being unclassifiable (6%) (Fig. 3). Similarly, the majority of DLBCL with *BCL2*-SH were GCB (74%), with the remaining cases distributed among MHG (14%), ABC (4%) and unclassified (8%). It is worth noting that the three cases of ABC-DLBCL with *BCL2*-SH lacked both *EZH2* and *GNA13* mutations that were nearly exclusively seen in GCB (MHG)-DLBCL.

### MHG-DLBCL with *MYC* translocation are enriched with *MYC* mutations that enhance its stability and transforming capacity

**Fig. 4:**
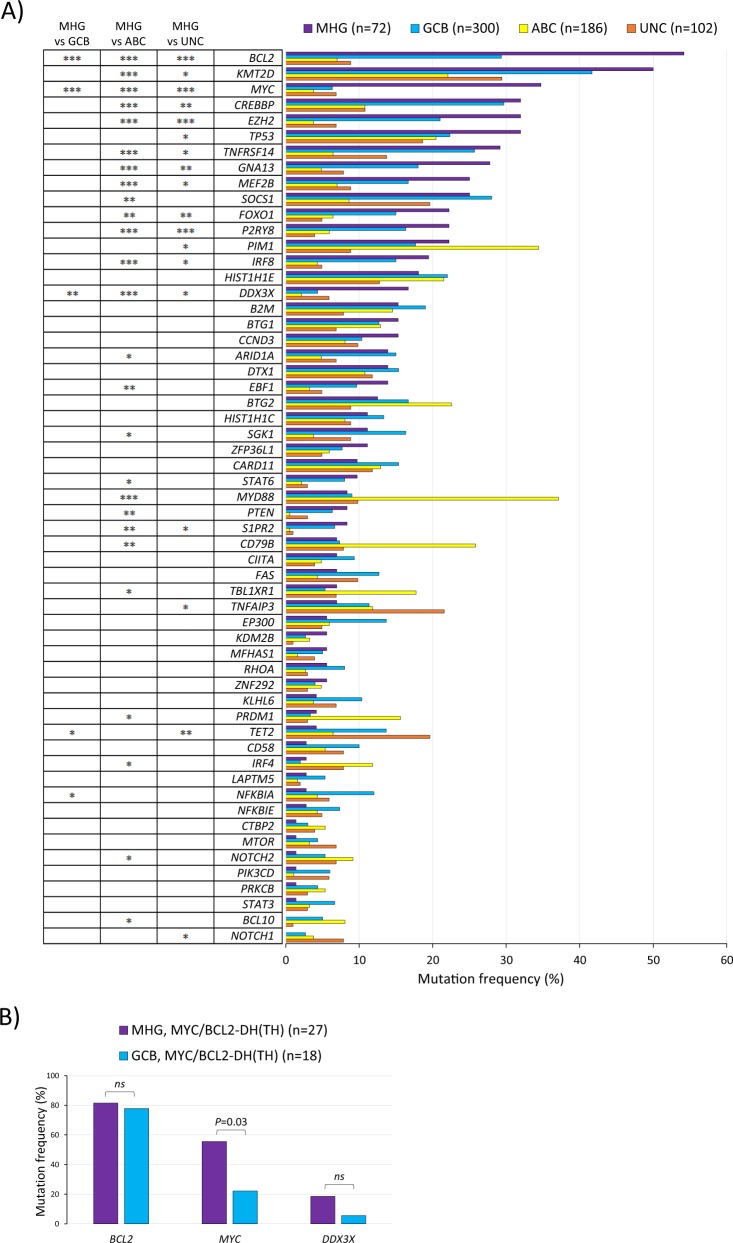
Mutation profile of MHG in comparison with the other molecular subtypes. **a** Comparison of mutation profile among MHG, GCB and ABC subtypes. The panel includes only the genes (*n* = 57) that had a mutation frequency ≥5% in at least one molecular subtype to make the figure legible. **b** Mutation comparison between MHG and GCB within the *MYC*/*BCL2*-DH(TH) group. Only genes showing a significant or apparent difference are included in the figure panel, with *BCL2* mutation included as a reference. Fisherʼs exact test was used to analyse the difference of mutation frequency between various groups with statistical significance indicated.

Our previous study showed that among patients with a *MYC* translocation, MHG-DLBCL had a significantly worse survival than GCB-DLBCL (non-MHG) [25]. To understand the genetic basis underlying MHG-DLBCL and its aggressive clinical behaviour, we compared the mutation profile among MHG, GCB and ABC subtype, and also between MHG and GCB within *MYC*/*BCL2* double-hit groups (Fig. 4). This revealed a significantly higher frequency of *MYC* mutations in the MHG group (Fig. 4b).

**Fig. 5:**
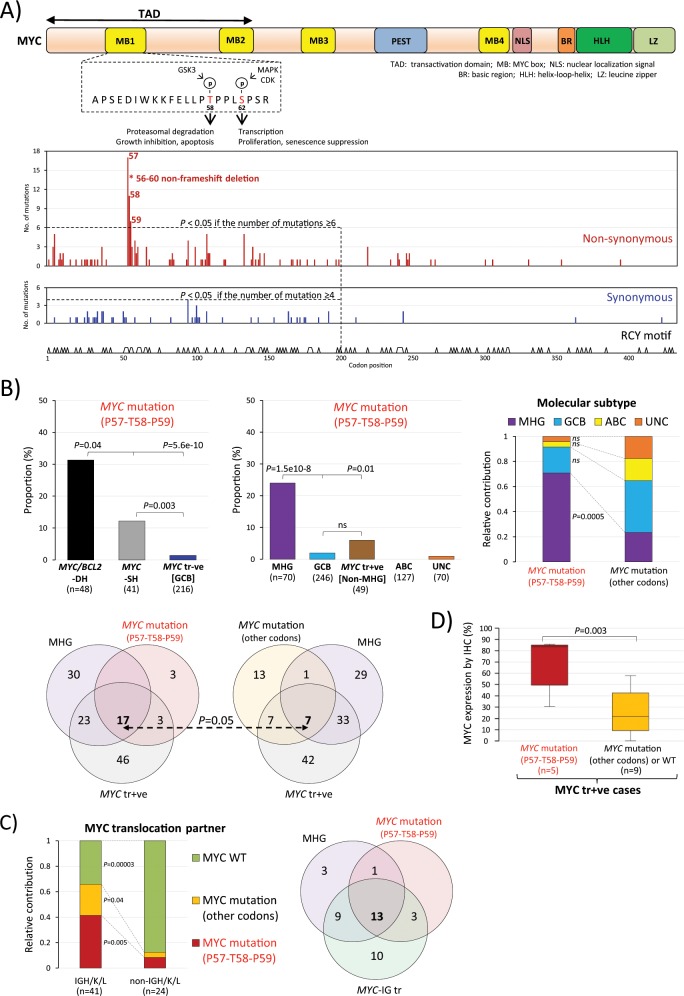
*MYC* pathogenic mutations and their relationship to molecular subtype and genetic changes in DLBCL. **a** Distribution of *MYC* variants with hotspot mutations clustered at the phosphorylation site (T58) and its neighbouring amino acids that are important for *MYC* degradation. *One case shows an in-frame deletion of codons 56–60 and all other mutations are missense changes. The codons 57, 58 and 59 hotspot mutations and the in-frame deletion are likely pathogenic and selected during lymphoma development. *MYC* variants are annotated according to transcript ENST00000377970.6 in keeping with the literature. **b** *MYC* hotspot mutations in codons 57, 58 and 59 are seen in a subset of cases with *MYC* translocation, more frequent in those with *MYC/BCL2-DH*, but are significantly enriched in MHG-DLBCL. **c** *MYC* hotspot mutations are more commonly seen in *MYC/BCL2-DH* DLBCL with *IG* gene as the *MYC* translocation partner, with a considerable overlap with MHG phenotype. **d** DLBCL with *MYC* mutation in codons 57–59 show a significantly higher MYC protein expression than those with *MYC* translocation, but lacking these pathogenic mutations. The MYC protein expression was investigated by immunohistochemistry, quantified using IHC Profiler Image-J software and presented as percentage of positive nuclear staining in lymphoma cells. Unpaired *t*-test was used to compare the two groups. MHG molecular high-grade, GCB germinal centre B-cell like, ABC activated B-cell like UNC, unclassified.

*MYC* is a known target of somatic hypermutation machinery, and as expected many of the *MYC* mutations were in the RCY-motif (R = A/G, Y = C/T), with their extent attenuating when further downstream from the promoter (Fig. 5a). In comparison with synonymous mutations, there was a significant enrichment of non-synonymous changes in codons 57, 58 and 59 (Fig. 5a). In addition, an in-frame deletion of codons 56–60 was seen in one case. These mutations are likely to be functional, pathogenic and selected during lymphoma development as they affect the phosphorylation site (T58) and its neighbouring amino acids, which are important for MYC protein degradation [43]. Several lymphoma derived MYC mutants, including P57S and T58A, have been previously shown to dramatically increase the half-life of MYC protein, and also confer increased transforming capacity [44, 45].

**Fig. 6:**
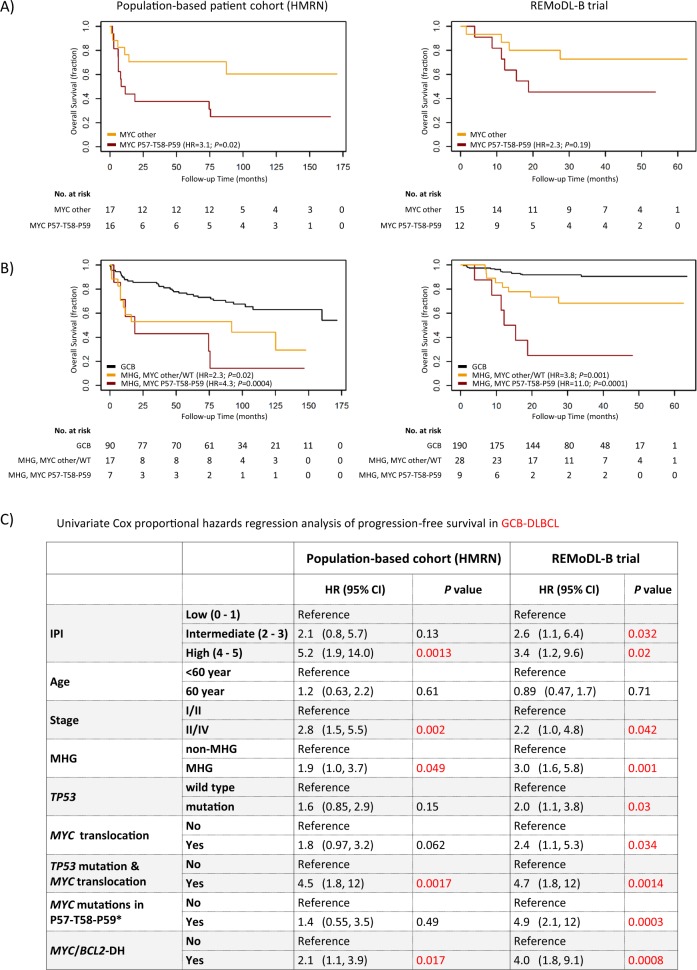
Prognostic value of *MYC* codons 57–59 mutations in DLBCL. **a** Differential impact on survival between *MYC* mutations in codons 57–59, and others. **b** MHG-DLBCL with *MYC* mutations in codons 57–59 show the worst overall survival in comparison with GCB-DLBCL. **c** Single variable Cox proportional hazards regression analysis of progression-free survival in GCB-DLBCL. *In multivariable analysis adjusting for MHG and *MYC*/*BCL2*-DH, *MYC* pathogenic mutations in codons 57–59 retain statistical significance in the REMoDL-B cohort, albeit not in the HMRN cohort.

These *MYC* hotspot mutations were seen in a subset of DLBCL with *MYC* translocation, more frequently in those with *MYC*/*BCL2*-DH, and the majority (74%) were MHG (Fig. 5b).  In contrast, cases with *MYC* non-synonymous mutations in other codons did not show any association with molecular subtype although occurred predominantly in those with *MYC*/*IG* translocation (Fig. 5b, c). *MYC* hotspot mutations were significantly more frequent in cases with *MYC*/*IG* (41%) than those with *MYC*/non-*IG* translocation (8%), and together had a considerable overlap with MHG phenotype (Fig. 5c). Cases with *MYC* hotspot mutations had a significantly higher MYC protein expression than those with other *MYC* mutations (Fig. 5d) as shown by immunohistochemistry and quantitative analysis of the scanned immunostained slides [25]. Finally, *MYC* pathogenic mutations had a potential adverse effect on patient survival (Fig. 6). Even within the MHG group, cases with *MYC* pathogenic mutations had significantly worse overall survival than those without these mutations in the REMoDL-B trial, and a similar trend was also seen in HMRN’s population-based cohort (Fig. 6a, b). In a separate analysis within cases with *MYC*/*BCL2*-DH irrespective of their MHG status, cases with *MYC* pathogenic mutations also had significantly worse overall survival than those without these mutations in the REMoDL-B trial, albeit not in HMRN’s population-based cohort (Fig. S4). In multivariable analysis adjusting for MHG and *MYC*/*BCL2*-DH, *MYC* pathogenic mutations retained statistical significance in the REMoDL-B group, albeit not in the HMRN cohort.

A low allelic frequency (4–5% AAF) of the above *MYC* pathogenic mutations was seen in three cases, likely representing a subclonal change. Among these three cases, two had molecular subtyping data and one was MHG.

### DLBCL with *MYC/BCL6-*DH or *BCL6-*SH, or *MYC*-SH are heterogeneous in their mutation profile and molecular subtype

There was no apparent mutation signature, nor biased molecular subtype associated with *BCL6* translocation with exception of *BCL10* and *NOTCH2* mutations which were significantly enriched in cases with *MYC/BCL6-*DH or *BCL6-*SH (Figs. 2a, 3 and S5). Similarly, there was no specific mutation signature in DLBCL with *MYC*-SH with the exception of high frequent *MYC* mutations. Cases with *MYC*-SH varied in their molecular subtype, nonetheless MHG (39%) and GCB (29%) accounted for the majority. Interestingly, 6 of the 11 MHG-DLBCL with *MYC*-SH had *TP53* mutations.

## Discussion

Using integrated analyses of chromosome translocation, somatic mutation profiling of a panel of 70 genes that are recurrently mutated in aggressive B-cell lymphoma, and molecular subtype in a large cohort of DLBCL, the present study made two novel observations.

First, we have provided several strands of evidence indicating that DLBCL with *MYC*/*BCL2*-DH and those with *BCL2*-SH are most likely derived from a low-grade FL or its precursor lesion. These include finding: (1) a cardinal mutation signature (*BCL2*, *KMT2D*, *CREBBP*, *EZH2* and *TNFRSF14*) associated with FL development; (2) a mutation profile (*MYC*, *GNA13*, *TP53*, *P2RY8, PIM1*, *CCND3*, *B2M*, *EBF1* and *S1PR2*) associated with FL high-grade transformation; and (3) frequent presence of a previous or concurrent FL in DLBCL with *BCL2* translocation.

We acknowledge the limitation of the relatively small gene panel used in the present study, and the above speculation needs to be confirmed by more comprehensive genetic profiling. Nonetheless, the speculation is supported by the finding that transformed FLs also show frequent *MYC* hotspot mutation [41]. In support of our study, the mutation signature associated with FL was also the characteristic change in high-grade B-cell lymphoma with *MYC* and *BCL2* translocations [46], and in the EZB or C3 genetic subgroup, which are enriched by *BCL2* translocation [23, 24].

Intriguingly, DLBCL with *MYC*/*BCL2*-DH had strong mutation signatures associated with FL, but less frequent evidence of a concurrent FL than those with *BCL2*-SH (28% vs 50%). Given the highly proliferative nature of DLBCL with *MYC*/*BCL2*-DH, these high-grade lymphoma cells may frequently efface the low-grade FL lesion, potentially leading to its underdetection. In addition, a single lymph node is commonly biopsied for histological diagnosis, increasingly needle core rather than excision biopsies. This would underestimate the true incidence of FL in patients with DLBCL. Alternatively, DLBCL with *MYC*/*BCL2*-DH may be derived from a precursor lesion, such as a common mutated precursor cell population. In fact, transformed FLs are more commonly (66–83%) derived from a common mutated precursor cell (CPC) population, in a process of divergent evolution [38–41]. The tissue compartment containing the CPC population is likely to be in situ follicular neoplasia (ISFN), the precursor lesion of FL, albeit this remains to be confirmed in future investigations. It is pertinent to speculate that *BCL2* translocation positive DLBCL could be similarly derived from an ISFN lesion, regardless of any evidence for parallel FL development.

Second, we have identified frequent *MYC* hotspot mutations that affect the phosphorylation site (T58) and its adjacent amino acids, which are critical for FBXW7 mediated proteasome degradation (Fig. 5a) [43]. Such lymphoma derived MYC mutants (T58A, P57S) have been shown to increase the half-life of MYC protein from 30 to 110 min, and also confer increased transforming capacity, but are defective in promoting apoptosis due to failure to activate BIM [44, 45, 47]. Thus, these hotspot mutations are likely to be pathogenic and selected during lymphoma development.

Although these *MYC* hotspot mutations were seen in a subset of cases with *MYC* translocation, they were predominantly in MHG.  This may explain, at least in part, the heterogeneous molecular subtype and clinical outcome of DLBCL with *MYC* translocation, including those with *MYC*/*BCL2*-DH. *MYC* hotspot mutations (pathogenic changes) were significantly more frequent in cases with *MYC/IG* than those with *MYC*/non-*IG* translocation (41% vs 8%, *P* = 0.005), potentially explaining in part why these cases showed a worse prognosis than those with non-IG gene as the *MYC* partner [48, 49]. Cases with *MYC* pathogenic mutations were significantly associated with higher MYC protein expression as assessed by immunohistochemistry and quantitative imaging analysis.  This could potentially explain in part the variability (20–100%) of MYC protein expression in tumour cells of *MYC* translocation positive DLBCL [50]. The above findings are also consistent with the observation that the adverse prognosis of the *MYC*/*BCL2*-DH group is primarily due to those with IG as MYC translocation partner [48, 51].

It is worth noting that the above *MYC* hotspot mutations were also seen at a subclonal level, albeit in only a few cases. This finding raises an interesting question about whether tumour cells carrying these mutations are more resistant to therapies, and could thus be enriched in resistant or relapsed disease. If this is the case, the clinical impact of these *MYC* mutations may be under-estimated in the present study, as the investigation was exclusively based on diagnostic tissue biopsies.

More recently, a second hotspot of tumour-associated *MYC* mutations was identified in codons 243–249 through meta-analysis of mutation data from Burkitt lymphoma [52]. These mutants (T244A, P245A) phenocopy the aforementioned mutations in their enhanced MYC protein stability, transforming capacity, and also defective BIM activation [52]. We did not observe any hotspot mutations in this region, but this could be due to the relatively small number of cases in the present study, or different mutation spectra between Burkitt and DLBCL. Mutation in codon 138 has also been suggested to enhance MYC protein stability and thus regarded as pathogenic change in DLBCL [53, 54]. Nonetheless, mutation in codon 138 was frequently accompanied by the change in codon 58, and its independent impact remains uncertain due to a very limited number of mutant cases identified [54].

In the 2017 WHO lymphoma classification, *MYC*/*BCL6*-DH DLBCL are included in the double-hit category, without any distinction from those with *MYC*/*BCL2*/*BCL6*-TH or *MYC*/*BCL2*-DH [55]. We show here that DLBCL with *MYC*/*BCL6*-DH are significantly different in their mutation profile and molecular subtype from those with *MYC*/*BCL2*/*BCL6*-TH or *MYC*/*BCL2*-DH. This observation is further supported by a recent study albeit based on a smaller cohort [46]. In fact, *MYC*/*BCL6*-DH DLBCL are highly heterogeneous in their molecular subtypes, indicating their diverse COO, notwithstanding the high prevalence of *NOTCH2* mutations [23, 24]. *BCL6* translocation is recurrently seen in both follicular and marginal zone lymphoma [56–59]. It would be interesting to explore whether *MYC*/*BCL6*-DH DLBCL also result from high-grade transformation of a low-grade lesion such as follicular or marginal zone B-cell lymphoma or their precursor lesions, and their heterogeneous molecular subtypes reflect their inherent features from their derived low-grade lesion. These heterogeneities, in addition to the small numbers of cases available for each study, may explain the disparate clinical outcomes reported for *MYC*/*BCL6*-DH DLBCL [14, 17, 18]. In light of this, *MYC*/*BCL6*-DH DLBCL should not be regarded as a single group, and their biology and clinical management need to be explored in the context of their respective molecular subtype, rather than within the double-hit category.

In summary, DLBCL with *MYC*/*BCL2*-DH harbour the characteristic mutation signatures that are associated with FL development and its high-grade transformation, suggesting their derivation from FL or its precursor lesion, probably following acquisition of a *MYC* translocation. Our study also identifies the novel association of MHG-DLBCL with *MYC* hotspot mutations that enhance its stability and transforming capacity, and further highlight the pathogenic role of these mutations and their clinical significance, beyond transcriptional deregulation as a result of translocation.

## Supplementary information

Supplementary material legend_clean

Figure S1

Figure S2

Figure S3

Figure S4

Figure S5

Supplementary tables
