# Supplementary material for: Distinct genetic changes reveal evolutionary history and heterogeneous molecular grade of DLBCL with MYC/BCL2 double-hit
Source: Leukemia. 2019 Dec 16;34(5):1329–41. doi: 10.1038/s41375-019-0691-6 (PMC7192846; doi:10.1038/s41375-019-0691-6)
Supplement: Supplementary file 1 — Supplementary material legend_clean [file 41375_2019_691_MOESM1_ESM.docx]

**SUPPLEMENTARY MATERIALS**

**Figure S1:** Similar mutation profile between DLBCL with *MYC*/BCL2/*BCL6*-TH and those with *MYC*/BCL2-DH.

**Figure S2:** A) Mutation loads in DLBCL according to translocation status. Upper panel includes mutation in all genes investigated; Lower panel excludes mutations in the gene affected by the respective translocation to eliminate bias as higher mutation activities are seen in the rearranged oncogenes. Unpaired t-test was used to compare MYC/BCL2-DH(TH) with the other groups. B) Comparison of mutations between *MYC*/*BCL2*-DH(TH) and *MYC*-SH DLBCL. Only genes showing significant or apparent differences are shown.

**Figure S3:**  Comparison of mutations between *BCL2* translocation positive DLBCL with and without a previous or concurrent follicular lymphoma. Only the mutations that are associated with follicular lymphoma development and its high-grade transformation are shown. The significant difference in *MYC* mutation frequency is due to a higher proportion of cases with *MYC* translocation in the group without previous or concurrent FL.

**Figure S4:** Prognostic value of *MYC* codons 57-59 mutations in DLBCL with *MYC*/*BCL2*-DH irrespective of their MHG status. Cases with *MYC* pathogenic mutations had significantly worse overall survival than those without these mutations in the REMoDL-B trial, albeit not in HMRN’s population-based cohort.

**Figure S5:** Mutation profile and molecular subtype according to *BCL6* translocation status.

**Table S1:** Gene panel for targeted sequencing in DLBCL.

**Table S2:** List of variants detected by targeted sequencing of a panel of 70 genes in DLBCL.
