# Supplementary figures and images for: Distinct genetic changes reveal evolutionary history and heterogeneous molecular grade of DLBCL with MYC/BCL2 double-hit
Source: Leukemia. 2019 Dec 16;34(5):1329–41. doi: 10.1038/s41375-019-0691-6 (PMC7192846; doi:10.1038/s41375-019-0691-6)

Figure S1

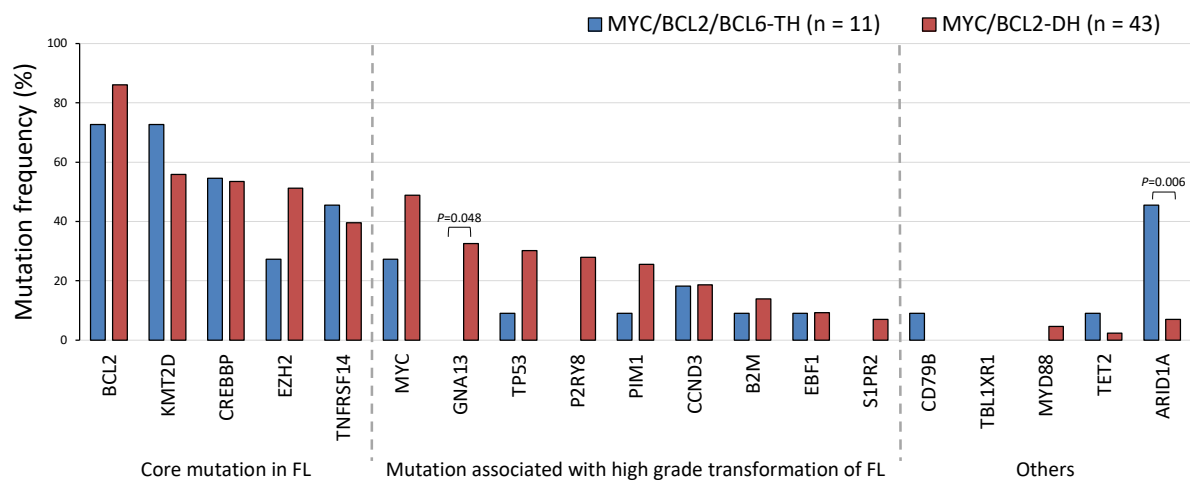

Supplement: Supplementary file 2 — Figure S1 [file 41375_2019_691_MOESM2_ESM.pdf]

Figure S2

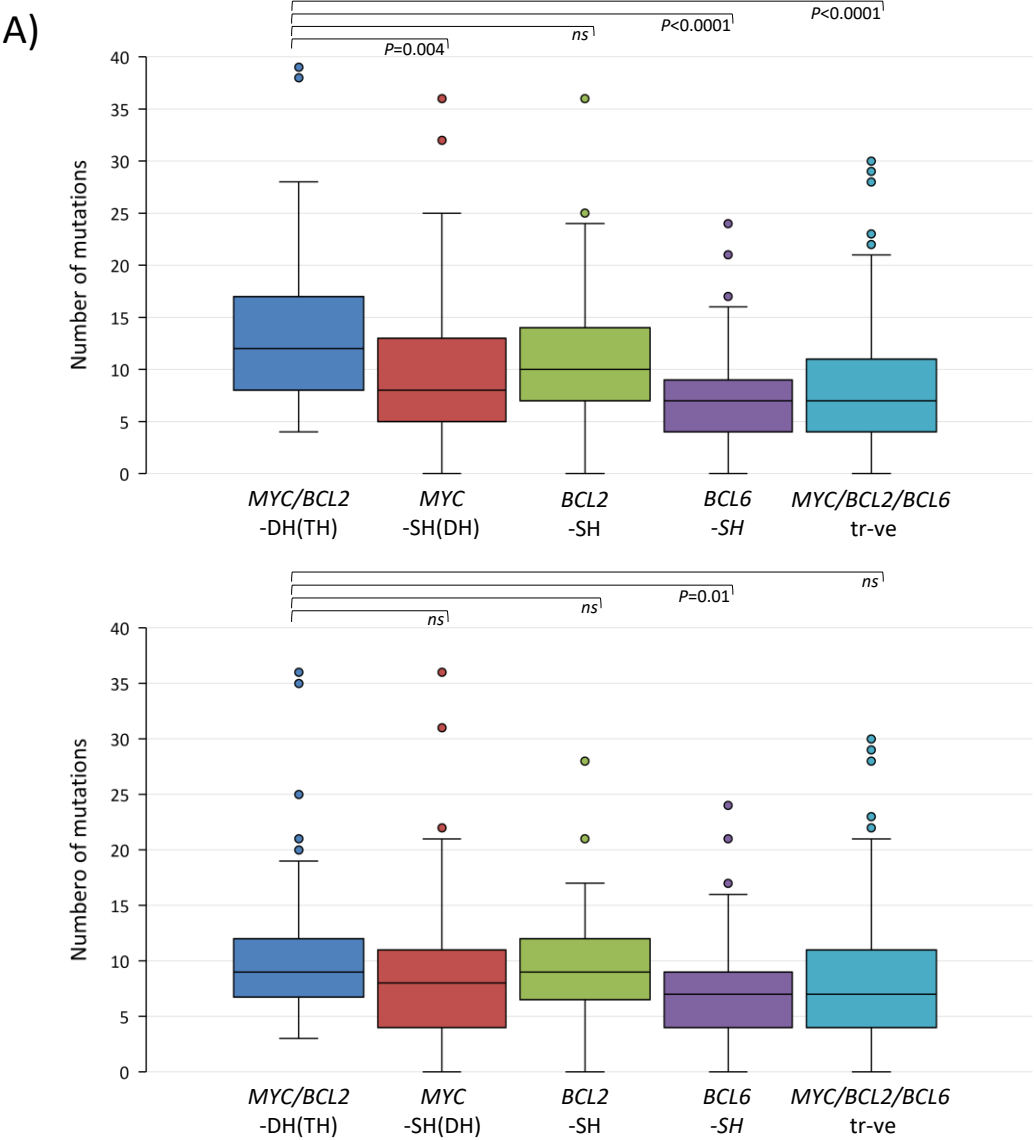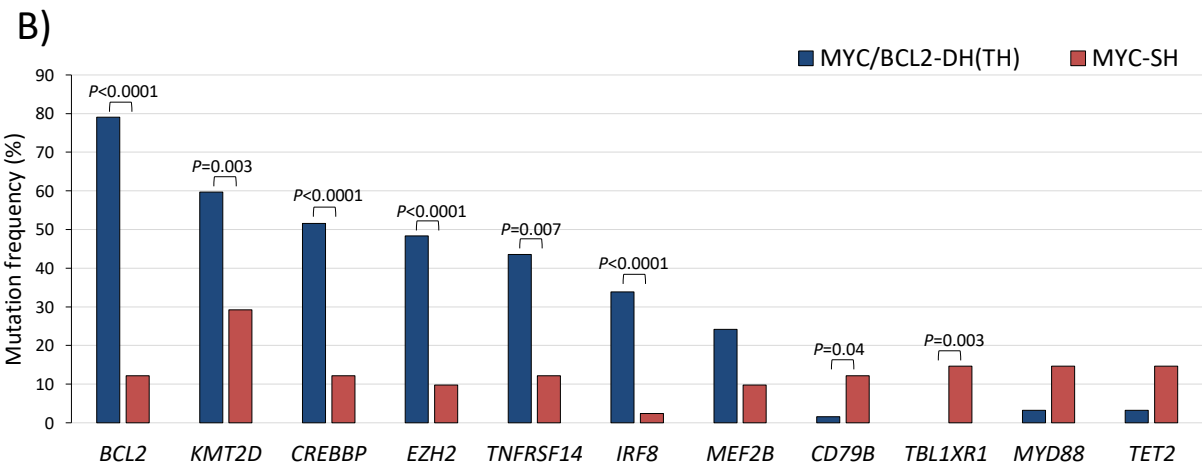

Supplement: Supplementary file 3 — Figure S2 [file 41375_2019_691_MOESM3_ESM.pdf]

Figure S3

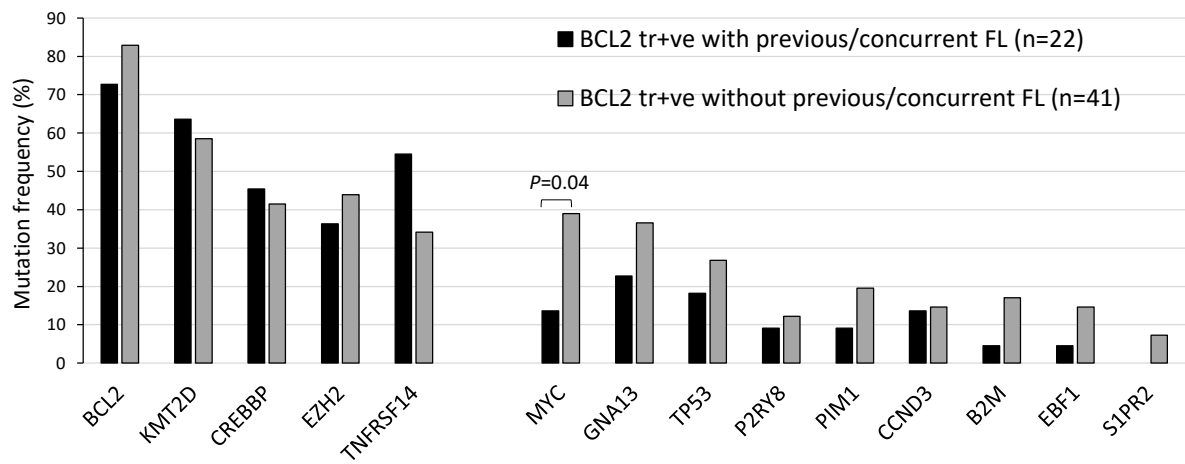

Supplement: Supplementary file 4 — Figure S3 [file 41375_2019_691_MOESM4_ESM.pdf]

Figure S4

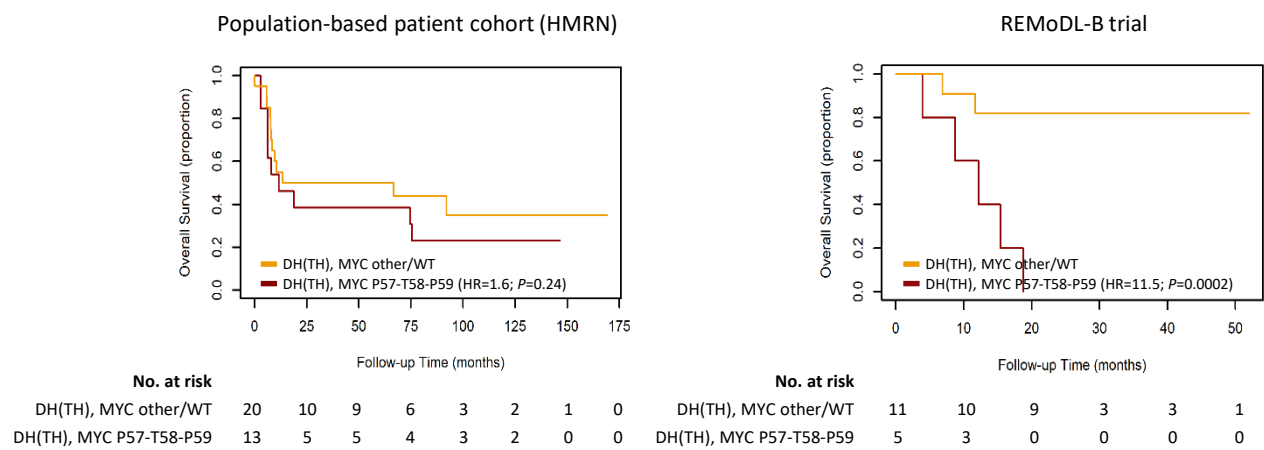

Supplement: Supplementary file 5 — Figure S4 [file 41375_2019_691_MOESM5_ESM.pdf]

Figure S5

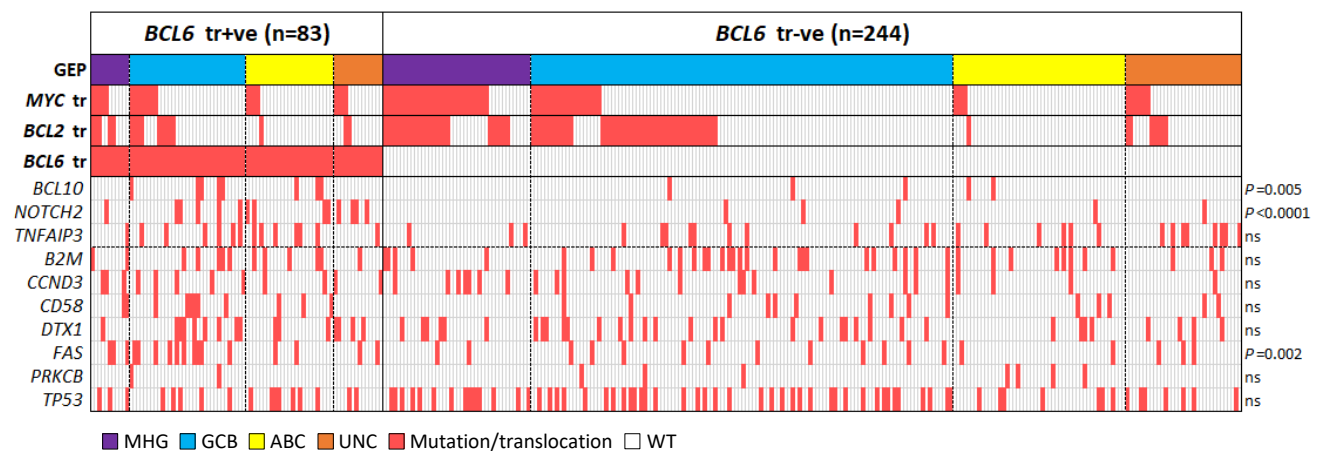

Supplement: Supplementary file 6 — Figure S5 [file 41375_2019_691_MOESM6_ESM.pdf]
